# Linking elevated HbA1c with atherogenic lipid profile among high risk cardiovascular patients at Qassim, Saudi Arabia

**DOI:** 10.6026/973206300200212

**Published:** 2024-03-31

**Authors:** Rihab Akasha, Saheem Ahmad, Rnada Abdeen, Sara Abulgasim, Heba Barnawi, Nagwan Elhussein, Rehab Hussien A, Dina Nawaf Alshammari, Nancy Elafandy, Sultan Alouffi

**Affiliations:** 1 Department of Clinical Laboratory Sciences,College of Applied Medical Sciences,University of Hail, Saudi Arabia

**Keywords:** HbA1c, Lipid Profile, Non-HDL cholesterol, Atherogenic Lipoproteins, LDL cholesterol

## Abstract

The relationship between glycated hemoglobin (HbA1c) and an atherogenic lipid profile which is associated with a higher risk of cardiovascular disease is of interest. A retrospective cross-sectional study was conducted on 83 participants aged between 14 and 77 years. Their venous blood was drawn to determine the HbA1c and fasting lipid profile including total cholesterol triglycerides and high-density lipoprotein cholesterol (HDL-C) low-density lipoprotein cholesterol (LDL-C) non-HDL cholesterol and the LDL/HDL ratio were also calculated. The correlations between HbA1c levels and these lipid profile parameters were analyzed. The study showed a significant correlation between HbA1c and LDL-C non-HDL-C and the LDL/HDL ratio. Although there was no significant difference in total cholesterol levels among all groups the levels of total cholesterol and HbA1c were positively correlated. HDL-C exhibited direct correlations with HbA1c there was no correlation between HbA1c and clinical characteristics except for age. Data shows that HbA1c can be used as a predictor of dyslipidemia in diabetic patients there is a significant correlation between HbA1c and an atherogenic lipid profile which highlights the importance of glycemic control in reducing the risk of cardiovascular disease.

## Background:

Diabetes is a leading cause of morbidity and death globally. It is significantly increases healthcare expenses as 425 million people worldwide had diabetes in 2017 and by 2040 that number is expected to reach 629 million cases. [1]. The total prevalence of Diabetes mellitus in the Kingdom of Saudi Arabia is 23.7% Saudis residing in cities had a higher prevalence of diabetes mellitus than the ones who lived in rural regions [2]. Diabetic people are more likely to develop cardio vascular disease (CVD) due to multiple risk factors associated with the physiological effects of diabetes on the cardiovascular system Diabetic patients also have a higher risk of myocardial infarction revascularization stroke and congestive heart failure. [3]. In addition patients with diabetes in particular type 2 diabetes mellitus (T2DM) are two to four times at higher risk of dying from cardiovascular diseases compared to non-diabetic patients [4]. Studies have found that an increased incidence of CVD in the general population is associated with hypertriglyceridemia low high-density lipoprotein cholesterol (HDL-C) high levels of total cholesterol (TC) and high levels of low-density-lipoprotein cholesterol (LDL-C) Numerous studies have confirmed that LDL-C is an independent predictor of cardiovascular CVD risk and it can be used for the assessment of the disease [5]. Recent studies have recognized elevated HbA1c as an independent risk factor for CVD in people with or without diabetes in addition to conventional risk factors such as dyslipidemia [6]. HbA1c is a type of hemoglobin that is chemically bound to sugar through glycation and it reflects the average plasma glucose concentration in patients over the 2-3 months prior to sample collection [7]. Hence, HbA1c is a crucial marker for assessing long-term glycemic control effectively capturing the overall glycemic history for patients. Further, levels of HbA1c exhibits a strong correlation with the likelihood of experiencing long-term complications associated with diabetes such as CVD [8]. As estimated there is 18% increases risk of CVD for diabetic patients for every 1% increase in HbA1c levels [9]. Known data shows that HbA1c is a potential biomarker for predicting dyslipidemia and CVD [10]. Therefore, it is of interest to link elevated HbA1c with atherogenic lipid profile among patients at Qassim, Saudi Arabia.

## Materials and Methods:

This cross-sectional retrospective study included 83 participants (45 male and 38 female) aged from 14 to 77 years. From these participants seven milliliters of fasting blood were drawn; five milliliters went into a plain tube for the lipid profile assay and two milliliters went into an EDTA tube for the HbA1c assay. Those in the plain tube were left to clot and retract for an hour and then the supernatant (serum) which was separated was centrifuged at 3000 rpm for five minutes at room temperature to determine the amounts of TC HDL-C LDL-C and TG. The EDTA samples were homogeneously blended for 5 minutes before the HbA1c concentration was determined. Analysis of TGs concentrations and HDL-C were performed using a Roche Cobas 6000 analyzer (Roche Diagnostics GmbH Mannheim Germany) using colorimetric or enzymatic assays following the fully validated assay procedures in routine use in the department of Clinical Biochemistry King Fahad Specialist Hospital Qassim Saudi Arabia.

The calculated LDL-C was estimated by using the Friedewald formula:

[LDL-C= (Cholesterol- HDL-C+TGs/2.2)].

Non-HDL-C was calculated as the following: Non-HDL-C = (TC) - (HDL-C).

HbA1c levels were determined using the boronate affinity chromatography method and reported in percentage (%).

## Statistics:

To determine the significance of the difference between the means of two groups of samples the data was expressed as mean ± SD. the Student unpaired t-test was used to compare groups with normal distribution and the Mann-Whitney test for non-Gaussian distribution. To conduct multiple comparisons the Bonferroni test was used. In addition Pearson's correlation test was performed to examine various correlations. Differences in significant were considered when the p-value was less than 0.05 (p < 0.05).

## Results:

**Figure 1:**
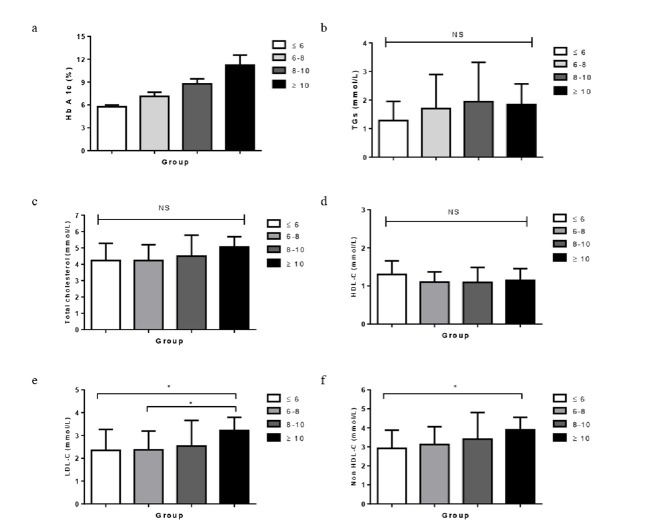
Serum concentrations of HbA1c (a), TGs (b), TC (c), HDL-C (d), LDL-C (e) and non HDL-C (f) in less than 6% group (white bar), 6-8 group (light grey bar), 8-10% (grey bar) and more than 10 % (black bar). Values are means ± SD (n = 24 for in less than 6% group, n = 26 for 6-8% group, n = 19 for 8-10% group and n = 14 for more than 10 % group). NS: not significant. *P<0.05 **P<0.01. ***P<0.001.

**Figure 2:**
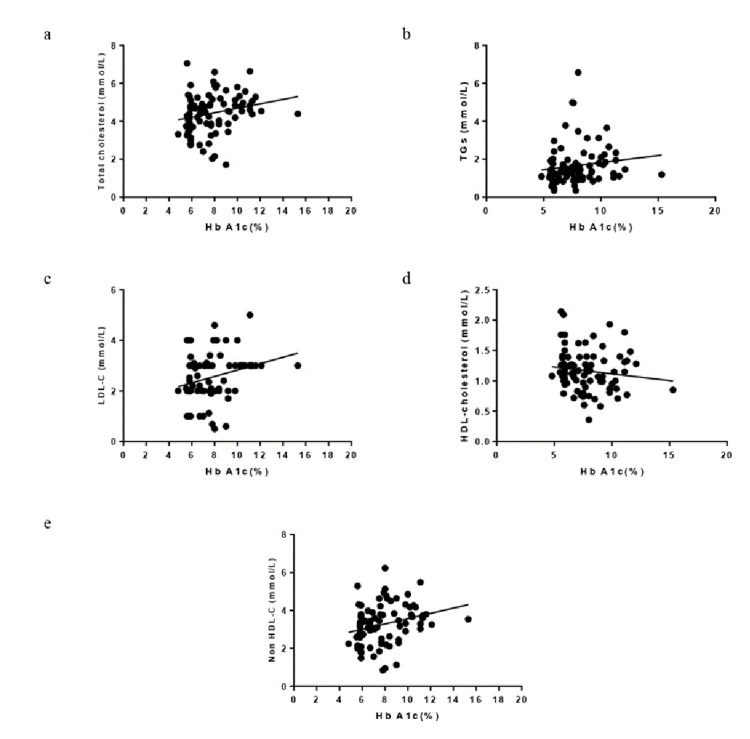
Correlation of Hb A1C versus total cholesterol (a), TGs (b), LDL-C (c), HDL-C (d) and non HDL-C (e) in all subjects.

Table 1 and Table 2 report the clinical and biochemical data of all subjects involved in this study. A total of 83 participants were selected with 45 (54.2%) males and 38 (45.8%) females and no differences in age were observed. Participants were divided into four groups based on their HbA1c levels: < 6% 6-8% 8-10% and > 10% (as shown in Figure 1a). In Figure 1b the mean serum concentration of TGs is displayed. Overall there was a tendency for individuals with higher HbA1c levels to have higher levels of TGs and total cholesterol despite the fact that the differences were not statistically significant. However the mean values of LDL-cholesterol and non-HDL cholesterol were significantly increased with increased HbA1c among all groups (as shown in Figure 1e and Figure 1f respectively). Additionally the mean values of LDL/HDL ratio were significantly increased with increased HbA1c among all groups with a significant correlation between HbA1c and LDL/HDL ratio (R2 = 0.271 P = 0.007) (as reported in Table 3). Table 4 displays the correlation between HbA1c and clinical characteristics in all subjects. Except for age there was no significant correlation between HbA1c and clinical characteristics. Age showed a significant correlation (R2 = 0.389 P =0.000) although there was no significant difference in total cholesterol levels among all groups there was a significant correlation between HbA1c and total cholesterol (R2 = 0.22 P = 0.0425) for all subjects (as displayed in Figure 1c). The correlation between HbA1c and TGs and HDL-cholesterol showed no significant relationship (as shown in Figure 2a and Figure 2b respectively). However the correlation between HbA1c and HDL-cholesterol was negative. HbA1c demonstrated a significant correlation with LDL-cholesterol (R2 = 0.274 P = 0.0122) (as displayed in Figure 2d). Furthermore it was found that HbA1c was positively and significantly related to non-HDL cholesterol (R2 = 0.2623 P = 0.0166) (as shown in Figure 2e).

## Discussion:

This study aimed to examine the relationship between HbA1c levels and lipid profile. The results showed a significant positive correlation between HbA1c and total cholesterol which is consistent with findings from previous studies [11] and also with recent study done in Jeddah City, Saudi Arabia [12]. This correlation suggests that managing dyslipidemia is crucial in preventing the progression of CVD and related complications. Notably both groups with HbA1c values of 6-8% and 8-10% had slightly lower levels of HDL-C. In an analysis of patients with high cardiovascular risk (CVR) or coronary heart disease (HD) equivalents low HDL-C was present in 66% reaching 79% in patients with controlled LDL-C regardless of statin therapy [13]. Individuals with an HbA1c level greater than 10% showed a significant increase in LDL-C levels compared to other groups. This is a concerning finding because LDL-C is a major risk factor for CVD and a target for current risk reduction strategies [14]. Many studies have established a clear link between LDL-C and CVD particularly CHD [15]. It has been hypothesized that even those with normal LDL-C levels may still develop CVD due to the increased atherogenic potential of sdLDL [16]. One study found that sdLDL-C concentrations are a better marker for assessing coronary heart disease risk than total LDL-C and could therefore be a new test for heart disease risk assessment [17]. Another study showed that elevated sdLDL concentrations are a significant marker of coronary artery disease risk in non-diabetic individuals [18]. This study has found that individuals with HbA1c levels above 10% have high levels of Non-HDL cholesterol. Non-HDL cholesterol is also significantly associated with HbA1c levels. This measurement is considered a useful tool for assessing CVR in individuals whose risk is not accurately identified by LDL cholesterol alone. Non-HDL cholesterol measures apo B-containing lipoproteins which provide information on atherogenic lipids [19]. Measuring non-HDL cholesterol is beneficial and cost-effective as it does not require a 12-hour fast which can be risky for hypoglycemia in T2DM patients [20]. Even if LDL cholesterol levels are at or below the NCEP goal or appear normal in T2DM increased non-HDL cholesterol levels have been reported to contribute to an increased risk of CVD [21]. The study also found a strong positive association between the LDL/HDL ratio and HbA1c. These findings are consistent with those of another study [22]; that found a substantial positive association between HbA1c TC TG LDL-C and LDL/HDL-C ratio. Managing dyslipidemia is recommended for various diseases such as diabetes and chronic kidney disease due to its strong association with CVD. Studies have shown that reducing total and LDL cholesterol levels significantly decreases the risk of CVD in the early stages of chronic kidney disease [23]. However it is important to consider the limitations of this study. The sample size was relatively low as the main focus was on metabolic investigation. Moreover the lack of data on medication types and patient adherence to medications for diabetes hypertension and dyslipidemia may limit the interpretation of the results. Lastly as the data was cross-sectional it is unclear whether there is a causal relationship between dyslipidemia and glycemic control.

## Conclusion:

Patients with high levels of HbA1c often have a profile of atherogenic lipoprotein which may contribute to the development of CVD and suggest a link between dyslipidemia and diabetes. HbA1c can also predict dyslipidemia in diabetic patients and early diagnosis of dyslipidemia can prevent CVD development. Non-traditional lipid tests like Non-HDL cholesterol can be useful in assessing the CVD risk. These findings highlight the need for further research on the role of lipids particularly lipoproteins in CVD risk for patients with diabetes.

## Figures and Tables

**Table 1:** Serum Lipid Profile of Subjects Group

**Parameter**	**Less than 6 % group (*n*= 24)**			**6-8 % group (*n*= 26)**			**8-10 % group (*n*= 19)**			**more than 10% group (*n*= 14)**		
	**Mean**	**SD**	**Range**	**Mean**	**SD**	**Range**	**Mean**	**SD**	**Range**	**Mean**	**SD**	**Range**
HbA1c (%)	5.75	0.24	4.8-5.9	7.13	0.55	6.1-7.9	8.79	0.64	8.0-9.8	11.2	1.31	10.0-15.3
TC (mmol/L)	4.22	1.05	2.7-7.05	4.22	0.96	2.03-6.1	4.5	1.3	1.7-6.6	5.05	0.63	4.4-6.6
TGs (mmol/L)	1.28	0.67	0.35-2.9	1.7	1.2	0.35-4.9	1.94	1.4	0.8-6.6	1.84	0.73	1.04-3.65
LDL-C (mmol/L)	2.35	0.91	1.0-4.0	2.49	0.75	1.0-4.0	2.54	1.1	0.5-4.6	3.21	0.85	3.0-5.0
HDL-C (mmol/L)	1.3	0.37	0.79-2.14	1.1	0.7	0.6-1.6	1.1	0.4	0.4-1.9	1.15	0.31	0.71-1.8
Non HDL-C (mmol/L)	2.92	0.96	1.5-5.3	3.12	0.94	0.85-4.9	3.4	1.4	0.95-6.2	3.9	0.65	3.03-5.5
LDL/HDL ratio	1.93	0.88	1.05-2.81	2.26	0.85	1.41-3.11	2.88	2.68	0.2-5.56	2.98	0.91	2.07-3.89
Data are mean ± SD

**Table 2:** Clinical Characteristics of Subjects

	**< 6 % group**	**6-8 % group**	**8-10 % group**	**>10% group**
**Number (Female)**	**24(9)**	**26(10)**	**19(10)**	**14(9)**
Mean age (years) Range (years)	29.5±9.1 14-46	54.2±17.1 35-70	52.2±16.1 19-74	50.1±19.7 15-77
Type 1 Diabetes	0	2	0	1
Type 2 Diabetes	4	14	11	8
Diabetes insipidus	3	0	0	1
Hypertension	2	0	0	1
Hypothyroid	5	3	2	0
Obesity	2	0	1	0
Others	8	7	5	3
Data are means ± SD

**Table 3:** Correlations between HbA1c and other biochemical tests in all subjects

**Parameter**	**Pearson correlation**	**P**
TC (mmol/L)	0.022	0.0425*
TGs (mmol/L)	0.1423	NS
LDL-C (mmol/L)	0.2741	0.0122*
HDL-C (mmol/L)	-0.1336	NS
Non HDL-C (mmol/L)	0.2623	0.0166*
LDL/HDL ratio	0.271	0.007**
*Statistically significance

**Table 4:** Correlations between HbA1c and clinical characteristics in all subjects

**Parameter**	**Pearson correlation**	**P**
Gender	0.194	NS
Age	0.389	0.000**
Type 1 Diabetes	-0.417	NS
Type 1 Diabetes	-0.38	NS
Diabetes insipidus	0.08	NS
Hypertension	-0.42	NS
Hypothyroidism	-0.712	NS
Obesity	0.083	NS
Others	-0.547	NS
*Statistically significance
